# Prevalence of Common Mental Disorders in a Rural District of Kenya, and Socio-Demographic Risk Factors

**DOI:** 10.3390/ijerph9051810

**Published:** 2012-05-09

**Authors:** Rachel Jenkins, Frank Njenga, Marx Okonji, Pius Kigamwa, Makheti Baraza, James Ayuyo, Nicola Singleton, Sally McManus, David Kiima

**Affiliations:** 1 Director, WHO Collaborating Centre (Mental Health), Institute of Psychiatry, King’s College London, P.O. Box 35, De Crespigny Park, London SE5 8AF, UK; 2 Consultant Psychiatrist, Upper Hill Medical Centre, Nairobi, Kenya; Email: fnjenga@africaonline.co.ke; 3 Consultant Psychiatrist, The Nairobi Hospital, Nairobi, Kenya; Email: marxokonji@wananchi.com; 4 Department of Psychiatry, University of Nairobi, Nairobi, Kenya; Email: pkigamwa@africaonline.co.ke; 5 Ministry of Health Survey Department, Nairobi, Kenya; 6 Mildmay International Kenya, Kisumu, Kenya; Email: jayuyo@mildmay.or.ke; 7 Director of Policy & Research, UK Drug Policy Commission, London N1 9NG, UK; Email: NSingleton@ukdpc.org.uk; 8 Research Director, National Centre for Social Research (NatCen), London EC1V 0AX, UK; Email: s.mcmanus@natcen.ac.uk; 9 Director of Mental Health, Ministry of Health, Nairobi, Kenya; Email: dmkiima@gmail.com

**Keywords:** epidemiology, Kenya, development

## Abstract

Association between common mental disorders (CMDs), equity, poverty and socio-economic functioning are relatively well explored in high income countries, but there have been fewer studies in low and middle income countries, despite the considerable burden posed by mental disorders, especially in Africa, and their potential impact on development. This paper reports a population-based epidemiological survey of a rural area in Kenya. A random sample of 2% of all adults living in private households in Maseno, Kisumu District of Nyanza Province, Kenya (50,000 population), were studied. The Clinical Interview Schedule-Revised (CIS-R) was used to determine the prevalence of common mental disorders (CMDs). Associations with socio-demographic and economic characteristics were explored. A CMD prevalence of 10.8% was found, with no gender difference. Higher rates of illness were found in those who were of older age and those in poor physical health. We conclude that CMDs are common in Kenya and rates are elevated among people who are older, and those in poor health.

## 1. Introduction

There is growing appreciation of the importance of mental health and mental disorders for social and economic development in Africa [1] and in the West [2] and there have been a number of epidemiological studies of mental disorders in Sub-Saharan Africa [3]. Kenya is one of the poorest countries in the world, ranked 144 out of 177 countries in the UN Development Programme’s ‘Human Development Report’ (HDR) for 2007, with one of the slowest economic growth rates in the region of 2.9% between 1996–2005 [4]. National absolute poverty declined from 52.3% in 1997 to 45.9% in 2005/2006, with gross national income per capita in 2005 at 520 USD, and up to 770 USD by 2008. The population is currently estimated to be 38 million and life expectancy is now 54 years. More than one in ten children die before the age of five, and four women out of every 1000 die in child birth [5]. The prevalence of HIV is 7.7% in women and 4% in men [6]. These socioeconomic and health challenges may impact on the mental health of the population. 

Previous epidemiological studies of mental health in Kenya using the Self-Reporting Questionnaire [7] and the Standardised Psychiatric Interview [8] have largely focused on people attending the district hospitals [9] and health centres [10,11]. There has been a study of the linkages between drug abuse, injection drug use and HIV/AIDS in Kenya [12], but there have been no previous epidemiological studies of mental disorder in the general population in Kenya. Therefore the current study aimed to determine the prevalence of common mental disorders at household level in a Kenyan rural community, and to examine associated risk factors. 

The research was conducted as part of a collaborative programme of work between the Kenya Ministry of Health, the UK Institute of Psychiatry WHO Collaborating Centre and the Kenya Psychiatric Association, and was funded by the UK Department for International Development. The overall collaborative programme of work included a detailed situation appraisal of context, needs, resources, provision and outcomes using the mental health country profile [13,14,15], a focus group study of sixty traditional healers in Maseno, exploring their views of mental illness, aetiology and treatment [16], a study of attitudes of primary care staff about mental illness [17]; previous surveys of primary care [18]; adaptation of the WHO primary care guidelines for Kenya, and development of mental health policy and strategy [19]. This epidemiological survey is important to provide a baseline of mental health needs in the country. 

## 2. Experimental Section

### 2.1. Site and Sample

The study sample was the 50,000 population living in Maseno. This is a rural agricultural area of western Kenya, on the edge of Lake Victoria. A one in fifty random sample was drawn of all 50,000 households in Maseno to give a projected sample of 1,000 households, using the 1999 Kenya Census conducted by the Government of Kenya in which all households had been enumerated. Each of the sampled households was then visited, all members recorded and on the same day one eligible person aged 16–65 selected at random for interview.

### 2.2. Implementation of the Survey

Efforts were made to use local capacity. Pencil and paper administration of the survey was coordinated by the Ministry of Health section for surveys. Interviews were conducted in 2004 by lay volunteer community health workers linked to primary care centres in Maseno. Interviewers received a brief orientation to the instrument and were trained in its use. Responses were recorded verbatim, scored and entered into an SPSS database. The CIS-R was administered by community health workers, supervised by a public health nurse working in Chulaimbo rural health training centre in Maseno. Informed consent was obtained from all respondents. 876 interviews were completed and the response rate was 87.6%.

### 2.3. Assessments

The survey gathered information about demographic and socioeconomic factors, common mental disorders (using the Clinical Interview Schedule–Revised (CIS-R)) [20], psychotic symptoms (using the Psychosis Screening Questionnaire (PSQ)) [21], and use of alcohol, drugs and tobacco [22,23]. Related articles will report the prevalence of psychotic symptoms and substance misuse in the population. All diagnostic categories of mental disorder included in the current paper were based on ICD 10 [24]. These ICD 10 diagnoses were derived by computer algorithms based on the combination of individual symptom scores, and were not allocated by the field interviewers. The CIS-R is a gold standard instrument for assessing psychopathology in community settings, which has been widely used in both rich and poor countries [25], and is designed to be used by lay interviewers. The instrument had not been used in Kenya before, so it was subjected to scrutiny by local mental health professionals and researchers and Ministry of Health survey officers to determine its suitability. It has been used recently in Tanzania [26]. The CIS-R uses an initial filter question about symptoms in the last month, and then asks frequency, duration and severity of each symptom in the last seven days. Calculation of prevalence is based on the last seven days. It provides diagnoses of depressive episode (mild, moderate or severe), obsessive compulsive disorder, panic disorder, phobic disorder, generalised anxiety disorder and mixed anxiety/depressive disorder. These diagnoses were the basis for an overall category of common mental disorder (otherwise non-psychotic disorder or neurosis). The numbers were not large enough for separate analyses of risk factors for individual diagnostic categories.

### 2.4. Analysis

Data was analysed using SPSS software for Windows Version 15. Chi squared (χ^2^) tests examined demographic and socio-economic differences between the areas. The raw data were weighted. The weights were calculated to take account of selection bias due to household size and to correct for the oversampling of Head of Household (HoH) and spouse, by weighting down those with a status of HoH/spouse. The final weighting variable did adjust for patterns of non-response in terms of differential probabilities of selection within households (*i.e.*, respondents living in households with more residents were weighted up due to the fact that they had a lower chance of selection), and adjusting the profile of the achieved sample to match that from Census. Odd ratios (ORs) with 95% confidence intervals (CIs) were calculated to determine significant associations with the presence of any CMD (any CMD is slightly different rate to 12+ score) and then “any CMD” was examined as the dependent variable in multi-variate logistic regression. 

### 2.5. Ethics Approval

Approval was granted by Mathari National Mental Hospital, Ministry of Health, Kenya, and Maudsley (SLaM), National Health Service (NHS) Foundation Trust.

## 3. Results

The response rate was 87.6%. The point prevalence of CMD in this sample was 10.8%, largely comprising mixed anxiety depression (6.1%), panic disorder (2.6%), generalised anxiety disorder (1.6%) and depressive episodes (0.7%; Table 1). Rates of illness were significantly higher with increasing age (*p* = 0.02) and presence of physical illness (*p* = 0.08; Table 2). Adjusted odds ratios for these variables are shown in Table 3.

**Table 1:** Prevalence of common mental disorders (CMDs) ^a^ in a community based sample in Maseno.

	n	%	Standard deviation (95%)
Total	876		
Any CMD	83	10.8	0.31
Specific CMDs			
Mixed anxiety and depression	48	6.1	0.24
Panic disorder	17	2.6	0.16
Generalised anxiety disorder	14	1.6	0.13
Depressive episode	9	0.7	0.08
Phobic disorder	3	0.3	0.05
Obsessive compulsive disorder	2	0.2	0.04

^a^ Any CMD and specific CMDs in the past seven days as measured by the Clinical Interview Schedule-Revised (CIS-R).

**Table 2:** Prevalence and unadjusted odds ratios for CMD by socio-demographic and health related factors.

	n	Prevalence of CMD %	Unadjusted odds ratios	CI (95%)	
Sex					
Male	292	10.9	1.0		
Female	584	10.8	0.98	(0.62, 1.55)	
Age group					
16–29	304	5.8	1.0		
30–64	562	14.7	2.8 ^a^	(1.25, 6.33)	
Marital status					
Married/cohabitating	684	11.9	1.0		
Single	66	8.1	0.64	(0.35, 1.18)	
Widowed	105	12.9	1.17	(0.43, 3.21)	
Relationship to head of household					
Head	456	8.5	1.0		
Spouse/partner	311	6.9	0.81	(0.41, 1.59)	
Son/daughter/other	86	11.3	1.37	(0.41, 4.54)	
Education					
None	112	13.4	1.0		
Primary	558	9.0	0.40	(0.09, 1.74)	
Secondary	146	7.5	0.39	(0.10, 1.63)	
Post secondary/current	43	11.6	0.74	(0.19, 2.80)	
Employment status					
None	116	7.1	1.0		
Farmer	535	11.8	1.70	(0.26, 11.17)	
Casual/wage worker	81	15.2	2.22	(0.20, 24.02)	
Trade/business	101	6.8	0.95	(0.11, 8.08)	
Type of home					
Permanent structure	169	14.1	1.0		
Semi-permanent	466	10.7	0.73	(0.23, 2.33)	
Temporary	233	8.1	0.55	(0.24, 1.23)	
Poor general health					
No	770	7.1	1.0		
Yes	102	37.6	8.1 ^b^	(1.96, 33.62)	

^a^*p* = 0.017; ^b^*p* = 0.008.

**Table 3:** Adjusted odds ratios for CMD.

	n	Adjusted odds ratios ^a^	CI (95%)	
Age group				
16–29	304	1		
30–64	559	2.47 ^b^	(1.46, 4.17)	
Poor general health				
No	761	1		
Yes	102	7.4 ^c^	(4.57, 12.00)	

^a^ variables identified as significant univariate predictors of CMD; ^b^ *p* = 0.001; ^c^ *p* = 0.000.

## 4. Discussion

This is the first epidemiological study of mental disorder in Kenya at the household level. It found a prevalence rate of 10.8%, with no gender difference. Significant risk factors were age and presence of physical illness. The prevalence rates found are higher than in neighboring Tanzania and also in Nigeria, but comparable to other recent studies in Sub-Saharan Africa—see Table 4. An earlier review suggested the prevalence rate of CMD in Africa ranges between 8% and 43% depending on the instrument used and population sampled [3], and wide variation is also found in other regions of the world (the lifetime rate of any disorder across 17 countries around the world was found to range between 12% and 47%) [27]. 

Recent studies in Nigeria found relatively low figures (e.g., any anxiety 4.1% and any mood disorder 1.3%; 5.2% overall depression [28]. Similarly, a recent study of CMD in Tanzania also found a relatively low rate of 3.1%) [26].

**Table 4:** Recent prevalence studies of common mental disorders in Sub-Saharan Africa.

Country	Author	Setting	Number	Measure	Prevalence
Lesotho	Hollifield *et al*. 1990	Rural	356	DIS	23.5% F
					14.7% M
Zimbabwe	Abas and Broadhead 1997	Urban	172	SSQ	15.7% F 1 month, 30.8% F 1 year
South Africa	Haavenaar *et al*. 2007	Periurban	209	SRQ	34.9% M and F
South Africa	Haavenaar *et al*. 2007	Rural	222	SRQ	27.0% M and F
South Africa	Hamad *et al*. 2008	Urban	257	CES-D	64.5% F, 50.4% M
Nigeria	Gureje *et al*. 2006	Probability sample of 8 states	4984	CIDI	12.1% life time; 5.8% 12 month prevalence
					M and F
Ethiopia	Tafari *et al*. 1991	Rural	2000	SRQ	11.2% M and F
Sudan	Rahim and Cederblad	Urban		SRQ/DSM III	16.6% M and F
South Africa	Stein *et al*. 2008	National Probability Sample	4433	CIDI	9.8% Mood disorders
					15.8% Anxiety disorders
					Lifetime prevalence
					M and F
Tanzania	Jenkins *et al*. 2010	Urban	899	CIS-R	3.1%

### 4.1. Risk Factors for CMD

In the current study, there was no difference in the prevalence of CMD between genders. In Nigeria, Gureje and colleagues also failed to demonstrate a difference between genders and, although consistent with Britain [29] and other parts of the world such as Brazil [30], higher rates among women have been found in Africa including Zimbabwe [31], Ethiopia [32], and South Africa [33,34,35]. Similar to findings in Tanzania [26] older age (30+ years) was associated with higher odds of CMD compared to people aged 16–24, while in Ethiopia rates were highest in people aged 35–44 [32], and 50–64 in Nigeria [36]. Patel and colleagues [37], in four low- and middle- income countries, and Lima and colleagues [30] in Brazil also found higher rates in older age groups. In Britain, the peak age groups were 50–54 for men and 45–49 for women [38]. 

Marital status was not significantly associated with disorder. The lack of significant association is consistent with recent reports from Nigeria [36] and South Africa [33], although previous studies have found higher rates in the widowed, separated and divorced in a number of developing countries [37]. 

Co-morbidity between mental illness and physical illness is a common finding in epidemiological surveys in rich, middle and low income countries, and Kenya is no exception [39]. 

## 5. Limitations

Although the response rate was satisfactory, we had a substantial amount of missing data resulting from the practical difficulties of maintaining adequate detailed supervision of a field exercise in rural Kenya with limited resource constraints. We also had higher than expected proportions of heads of household, indicating that sometimes the household members selected for interview had not been at random from an age ordered list, but rather preferentially of head of household. 

The missing data was largely in the later sections of the survey, such that we are unable to report on disability, life events, social networks, income and debt. In the earlier sections of the survey, missing data was less of a problem, and on exploring the data set with and without cases which had missing data, we found that the distribution of the CIS-R score was essentially similar using both approaches. Excluding cases with missing data resulted in a slightly higher prevalence rate of over 11%.

## 6. Conclusions

The prevalence of CMD in Kenya of 10.8% makes it a significant contributor to the overall public health burden and, in a country where there is only 1 psychiatrist per 500,000 population, makes integration of mental health into primary care a crucial task [40,41]. 
